# Intercostal artery embolization and the risk of spinal cord ischemia: Importance of identifying the Adamkiewicz artery—A report of two cases

**DOI:** 10.1016/j.radcr.2026.05.040

**Published:** 2026-06-06

**Authors:** Ryoki Semba, Tetsuya Katsumori, Hiroshi Miura

**Affiliations:** Department of Radiology, Saiseikai Shiga Hospital, Ohashi 2-4-1, Ritto, Shiga 520-3046, Japan

**Keywords:** Hemothorax, Intercostal artery, Rib fracture, Transcatheter arterial embolization, Adamkiewicz artery, Trauma

## Abstract

Intercostal artery embolization is a useful and minimally invasive procedure for traumatic hemothorax. However, the presence of the Adamkiewicz artery (AKA), which may originate from the intercostal artery, represents a critical risk factor for spinal cord ischemia during intercostal artery embolization. Here, we report two cases of traumatic hemothorax caused by intercostal artery injury that were successfully treated with transcatheter arterial embolization. The AKA was angiographically identified during the procedure in Case 1, whereas it was not recognized in Case 2, as it was located at the edge of the irradiation field. Despite this, embolization was successfully performed without non-target embolization of the AKA, and hemostasis was achieved without neurological complications. The identification of the AKA is crucial because inadvertent embolization may lead to serious spinal cord ischemia. Our cases emphasize the importance of evaluating the AKA during intercostal artery embolization.

## Introduction

Transcatheter arterial embolization (TAE) is an established and minimally invasive treatment for traumatic hemorrhage [1]. However, when applied to intercostal artery injury, a critical but potentially underrecognized risk arises. The Adamkiewicz artery (AKA), which may originate from the intercostal artery, supplies the anterior spinal artery, and inadvertent embolization can result in serious spinal cord ischemia [2,3]. The risk is well recognized in procedures such as thoracic endovascular aortic repair, spinal vascular interventions, and bronchial artery embolization. However, despite its clinical importance, this issue has not been sufficiently emphasized in the existing literature of intercostal artery embolization [[4], [5], [6], [7]].

Herein, we report two cases of traumatic hemothorax successfully treated with embolization of the intercostal artery from which the AKA arose, highlighting the importance of recognizing this critical anatomical structure.

## Case report

### Case 1

**Fig. 1:**
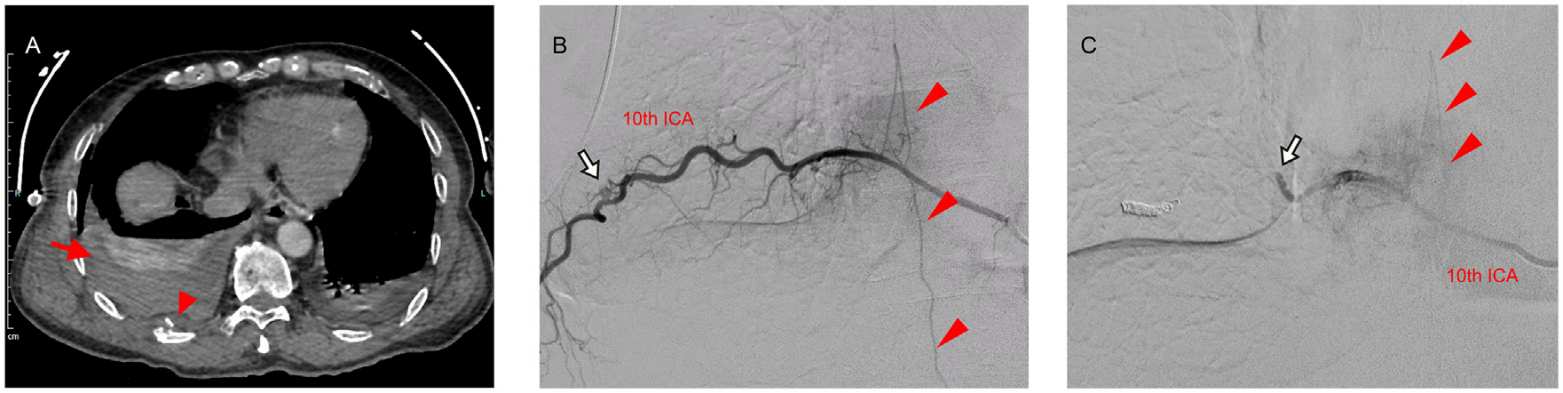
**(A)** Contrast-enhanced computed tomography (CT) obtained 3 days post-injury demonstrated a hemothorax (arrow) and a rib fracture (arrowhead) with no contrast medium extravasation. (B) Selective angiography of the right 10th intercostal artery revealed a small pseudoaneurysm (arrow) in the distal segment at the rib fracture level. The Adamkiewicz artery (arrowheads) originates from the proximal portion of the artery. (C) Embolization using metallic coils was successfully performed. Angiography after embolization demonstrated complete occlusion of the artery (arrow). The Adamkiewicz artery (arrowheads) is patent.

A 65-year-old man was transported to the emergency department after his car veered into a drainage ditch while he was driving. On arrival, he was in hemorrhagic shock. Emergent computed tomography (CT) demonstrated multiple injuries, including pulmonary contusions; hemothorax; multiple rib fractures; and fractures of the right femur, left humerus, and the 5th lumbar vertebral body. He was managed conservatively with fluid resuscitation, including blood transfusions. However, 3 days after the injury, he developed sudden shock accompanied by severe chest pain. Contrast-enhanced CT revealed a right hemothorax and rib fracture (Fig. 1A). Although CT imaging revealed no extravasation of contrast medium, there was a rapid increase in hematoma drainage from the chest tube. Because initiation of surgical hemostasis was expected to be delayed, embolization was attempted. Selective angiography of the right 10th intercostal artery revealed a small pseudoaneurysm in the distal segment at the level of the rib fracture, consistent with the source of bleeding that caused hypotension. The AKA, originating from the proximal portion of the right 10th intercostal artery, was identified, and a microcatheter was advanced to its distal portion. TAE was then successfully performed using micro metallic coils (Fig. 1B). Post-TAE angiography demonstrated complete occlusion of the artery with preservation of the AKA (Fig. 1C). Subsequently, active bleeding into the thoracic cavity decreased, and hemodynamic stability was achieved. The patient was discharged 60 days following the injury.

### Case 2

**Fig. 2:**
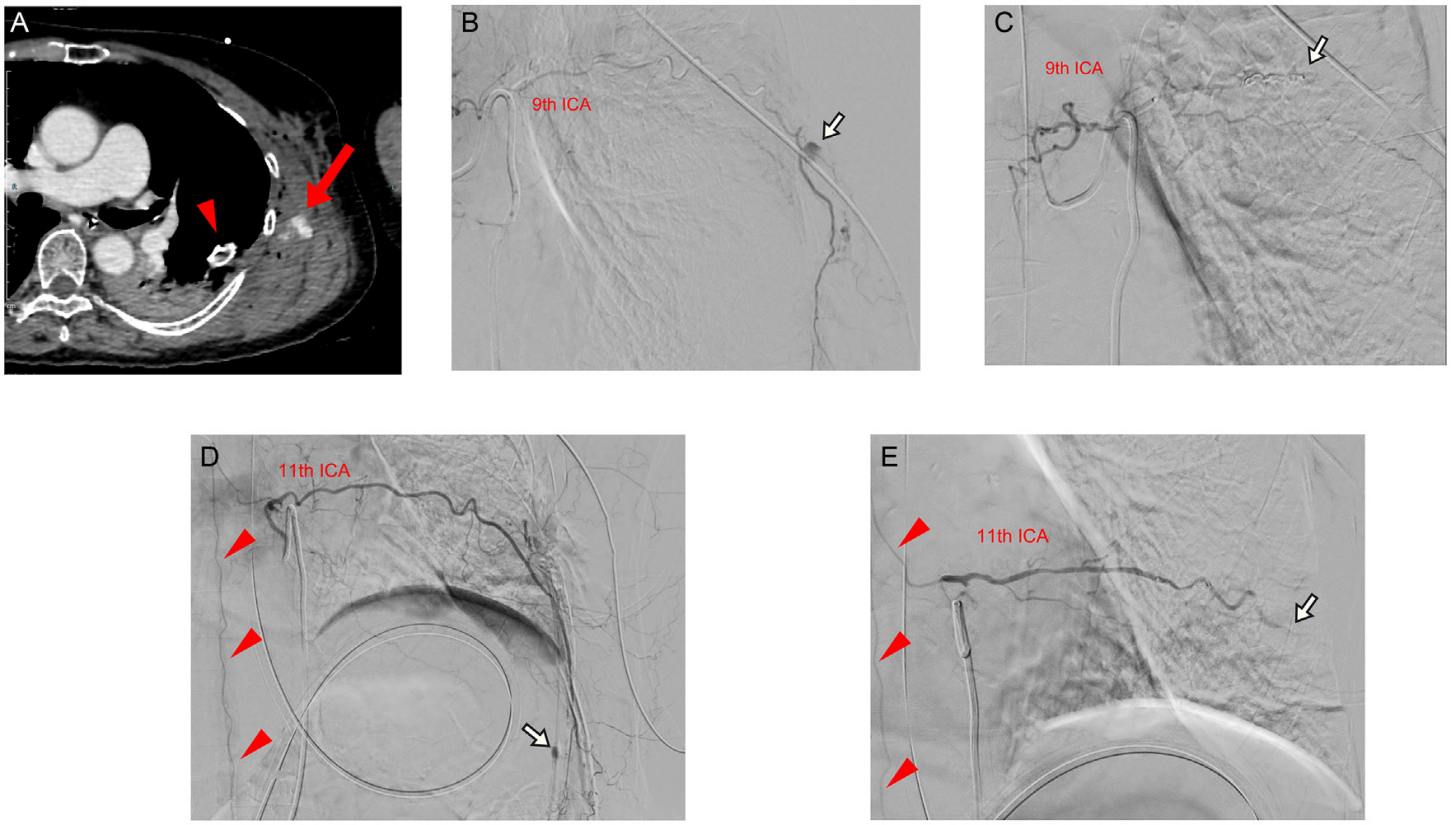
(A) Contrast-enhanced computed tomography (CT) after injury showed an extravasation of contrast media (arrow) from the chest wall. An arrowhead represents an aspiration tube inserted into the thoracic cavity for the left hemothorax. (B) Angiography revealed an extravasation (arrow) from the left 9th intercostal artery in the mid-portion at the rib fracture level. No Adamkiewicz artery was observed. (C) Angiography following embolization using gelatin sponge particles and metallic coils revealed complete occlusion of the artery (arrow). (D) Angiography revealed an extravasation (arrow) of contrast media from the left 11th intercostal artery in the mid-portion at the rib fracture level. The Adamkiewicz artery (arrowheads) originates from the proximal portion of the artery, but the vessel was not recognized at the time of the procedure. (E) Angiography after embolization with gelatin sponge particles and metallic coils showed complete occlusion of the artery (arrow), while the Adamkiewicz artery (arrowheads) remains patent.

A 72-year-old woman was involved in a motor vehicle collision and was hemodynamically unstable on arrival at the emergency department. Contrast-enhanced CT demonstrated pulmonary contusions, left hemothorax, multiple rib fractures, a pelvic fracture, and extravasation of contrast media from the chest wall, suggestive of active bleeding (Fig. 2A). There was a rapid increase in hematoma drainage from the chest tube. Despite initiating conservative management, such as fluid resuscitation, her hemodynamics remained unstable. TAE was attempted because surgical hemostasis was anticipated to require considerable time. Angiography revealed extravasation from the left 9th and 11th intercostal arteries in the mid-portion at the level of the rib fracture (Fig. 2B and 2D). Since the AKA was not identified, the left 9th intercostal artery was successfully embolized using gelatin sponge particles and metallic coils. Post-TAE angiography demonstrated complete occlusion of the target arteries (Fig. 2C). In contrast, selective angiography of the left 11th intercostal artery revealed that although the AKA originated from the proximal portion of the left 11th intercostal artery, the vessel was not recognized during the procedure as it was located at the edge of the irradiation field. Despite this, a microcatheter was advanced into the distal segment of the vessel, and TAE was successfully performed using gelatin sponge particles and metallic coils. Post-TAE angiography confirmed complete occlusion of the target artery while preserving the patency of the AKA (Fig. 2E). Following the conservative management, her hemothorax resolved, her vital signs improved, and she was discharged 52 days after the injury.

## Discussion

The present case report demonstrates that although intercostal artery embolization is an effective treatment for traumatic hemothorax [4,6,8], it carries a critical risk when the AKA is involved [2]. The AKA was identified on angiography during the procedure in Case 1, whereas it was not recognized during the procedure in Case 2, as it was located at the edge of the irradiation field, although it was retrospectively visible. Fortunately, embolization was successfully performed without non-target embolization of the AKA, and hemostasis was achieved without neurological complications. This vessel may not be recognized in clinical settings, potentially leading to serious spinal cord ischemia due to non-target embolization. Therefore, the identification and preservation of the AKA are essential in all intercostal artery embolization cases. Hence, the irradiation field of angiography is required to include not only the intercostal artery lesion but also the AKA. Further, embolization should be performed while simultaneously visualizing the target lesions and the AKA using angiography and/or fluoroscopy.

Spinal cord ischemia is the most serious complication of TAE for intercostal arteries, leading to paraplegia [2]. This adverse event is caused by non-target embolization of the AKA, which may originate from the proximal portion of the intercostal artery [2,3]. To avoid this complication, operators should confirm the presence or absence of the AKA on selective angiography of each intercostal artery and/or contrast-enhanced CT before performing TAE, although angiographic findings may occasionally differ from CT findings, as illustrated in Case 1 [3,7]. The catheter should then be advanced into the distal portion of the intercostal arteries to the AKA, and the migration of embolic agents into the AKA must be prevented [5,7].

The choice of embolic agents is closely related to AKA preservation. Metallic coils are suitable for embolizing the target lesion of the intercostal artery because of a lower possibility of migration into the AKA [5,7] (Case 1). When the AKA is absent from the intercostal artery, gelatin sponge particles or glue may be used [5,8], as demonstrated by embolization of the 9th intercostal artery in Case 2 (Fig. 2B and 2C). However, when the AKA originates from the intercostal artery, gelatin sponge particles or glue may not always be safe because they can be potentially refluxed and migrated into the AKA during the procedure [2,5]. In such cases, as demonstrated by the 11th intercostal artery in Case 2 (Fig. 2D and 2E), careful embolization is required to prevent reflux and migration of embolic agents into the AKA. The selection of a suitable embolic agent and the adoption of an appropriate technique must be tailored to each individual case.

Intercostal artery embolization is a useful procedure for traumatic hemothorax; however, thoracotomy or video-assisted thoracic surgery remains the first-line option for patients with hemodynamic instability or ongoing bleeding [9]. However, TAE can be indicated for patients who are hemodynamically stable or stabilized, or in those in whom surgical intervention is high risk due to age, comorbidities, or coagulopathy [6,9]. Further, this procedure is indicated for spontaneous non-traumatic bleeding from the intercostal artery [10].

In conclusion, intercostal artery embolization carries a critical risk of spinal cord ischemia when the AKA is involved. Failure to recognize this vessel may lead to serious neurological complications. Therefore, careful identification and preservation of the AKA should be considered essential during all intercostal artery embolization procedures.

## IRB approval

Institutional review board in the institution approved the publication of this case.

## Patient consent

Written informed consent was obtained from each patient for the retrospective use of medical data and for publication of this case report.
